# Anakinra Pilot – a clinical trial to demonstrate safety, feasibility and pharmacokinetics of interleukin 1 receptor antagonist in preterm infants

**DOI:** 10.3389/fimmu.2022.1022104

**Published:** 2022-10-27

**Authors:** Elys A. Green, David Metz, Robert Galinsky, Rebecka Atkinson, Elizbeth M. Skuza, Megan Clark, Alistair J Gunn, Carl M. Kirkpatrick, Rod W. Hunt, Philip J. Berger, Claudia A. Nold-Petry, Marcel F. Nold

**Affiliations:** ^1^ Department of Paediatrics, Monash University, Melbourne, VIC, Australia; ^2^ The Ritchie Centre, Hudson Institute of Medical Research, Melbourne, VIC, Australia; ^3^ Monash Newborn, Monash Children’s Hospital, Melbourne, VIC, Australia; ^4^ Monash Children’s Hospital, Melbourne, VIC, Australia; ^5^ Murdoch Children’s Research Institute, Melbourne, VIC, Australia; ^6^ Monash Institute for Pharmaceutical Sciences, Monash University, Melbourne, VIC, Australia; ^7^ Faculty of Pharmacy and Pharmaceutical Science, Monash University, Melbourne, VIC, Australia; ^8^ Department of Physiology, The University of Auckland, Auckland, New Zealand

**Keywords:** preterm infant, inflammation, anti-inflammatory therapy, interleukin 1 receptor antagonist, clinical trial, translational research, drug repurposing, protocol anakinra pilot

## Abstract

**Background:**

Bronchopulmonary dysplasia (BPD), its complication pulmonary hypertension (BPD-PH) and preterm brain and gut injury lead to significant morbidity and mortality in infants born extremely prematurely. There is extensive evidence that the pro-inflammatory cytokine interleukin 1 (IL-1) plays a key role in the pathophysiology of these illnesses. Two decades of clinical use in paediatric and adult medicine have established an excellent safety and efficacy record for IL-1 blockade with IL-1 receptor antagonist (IL-1Ra, medication name anakinra). Building on robust pre-clinical evidence, the Anakinra Pilot trial aims to demonstrate safety and feasibility of administering anakinra to preterm infants, and to establish pharmacokinetics in this population. Its ultimate goal is to facilitate large studies that will test whether anakinra can ameliorate early-life inflammation, thus alleviating multiple complications of prematurity.

**Methods and analysis:**

Anakinra Pilot is an investigator-initiated, single arm, safety and feasibility dose-escalation trial in extremely preterm infants born between 24 weeks 0 days (24^0^) and 27^6^ weeks of gestational age (GA). Enrolled infants will receive anakinra intravenously over the first 21 days after birth, starting in the first 24 h after birth. In the first phase, dosing is 1 mg/kg every 48 h, and dosage will increase to 1.5 mg/kg every 24 h in the second phase. Initial anakinra dosing was determined through population pharmacokinetic model simulations. During the study, there will be a interim analysis to confirm predictions before undertaking dose assessment. Anakinra therapy will be considered safe if the frequency of adverse outcomes/events does not exceed that expected in infants born at 24^0^-27^6^ weeks GA.

**Clinical Trial Registration:**

https://clinicaltrials.gov/, identifier NCT05280340.

## Introduction

Neonatal care has dramatically improved the survival of extremely premature newborn infants over the past 20 years. However, as a result, we face the high price of a steadily rising incidence in a number of morbidities, in virtually all of which inflammation plays a crucial pathophysiological role (1–5). The potent pro-inflammatory cytokine interleukin 1 (IL-1) is a key mediator of these inflammatory processes, as described below. Common examples of such inflammation-driven morbidities are bronchopulmonary dysplasia (BPD) (5), pulmonary hypertension associated with BPD (BPD-PH) (6), cerebral diffuse white matter injury (dWMI) and necrotising enterocolitis.

BPD is a chronic lung disease that can have devastating short- and long-term consequences, including impaired pulmonary and cardiovascular health and adverse neurodevelopmental outcomes (7, 8). BPD has become the most common chronic cardiopulmonary disease in infants, and, together with its gravest complication, BPD-PH, now represents a major burden for affected babies, their families, and health services alike (9). The costs of treating BPD are immense, and were estimated at US$1.7 billion per year in the USA alone in 2009 (10). Recognising that BPD and BPD-PH are inflammatory diseases, the American Academy of Pediatrics (AAP) issued an urgent call for new anti-inflammatory therapies for BPD in 2006 (11). With no safe and effective therapy forthcoming, BPD remains one of the greatest unmet challenges neonatology faces today; hence, the AAP renewed its call to escalate research efforts into the development of new anti-inflammatory strategies to offer a brighter prospect to the tiny patients and their families in 2014 (12).

Yet another morbidity of prematurity that results from a combination of systemic and local inflammation is diffuse white matter injury of the brain (dWMI). dWMI is defined by inflammation, scarring and loss/impaired development of the white matter, and it affects up to 40% of extremely preterm infants (2, 3), resulting in a variety of neurocognitive impairments that manifest in early childhood and persist into adulthood (13). In extremely preterm infants, the cerebral white matter, which contains axons that are insulated by myelin to facilitate rapid conduction of action potentials within and between neurons, is undergoing a period of rapid development and change. The precursor oligodendrocytes that populate the white matter and are ultimately responsible for ensheathing axons with myelin, are exquisitely sensitive to inflammation (14). Inflammatory damage to the vulnerable pre-oligodendrocytes results in dWMI with impairment of myelination, and ultimately neurodevelopmental impairment. Recent evidence has demonstrated that inflammatory white matter injury can persist into the postnatal period for up to 21 days (15).

Despite substantial advances in neonatal medicine over the past three decades, there is still no safe and effective therapy to prevent or at least treat inflammation-driven cardio-respiratory and cerebral injury in preterm infants. The antecedents of BPD (5, 16), BPD-PH (4, 6), and dWMI (2) include oxygen toxicity, fetal and postnatal infection or damaging mechanical ventilation, whose effects are mediated through a common pathway of inflammation. IL-1 is a key pathophysiological culprit in this process; it is highly associated with adverse outcomes in clinical cohorts (17–19), and its pathogenic role has been confirmed in a variety of clinically relevant models of BPD and BPD-PH in neonatal mice, piglets and rats, as reviewed in (20, 21), and in a model of inflammation-induced dWMI in fetal sheep (1, 22).

Interleukin-1 receptor antagonist (IL-1Ra) is an endogenous cytokine that binds to the receptor by which IL-1α and IL-1β exert their pro-inflammatory activities, thus competitively inhibiting their activity. Anakinra is a recombinant protein that is nearly identical to endogenous IL-1Ra. In rodent, piglet and sheep models of early life disease, treatment with exogenous IL-1Ra alleviated the loss of alveolar and pulmonary vascular tissue that characterises BPD, and the accompanying systemic and local inflammation (5, 6). These data are supported by reports in infants and animals, which show that there can be an imbalance between IL-1 and endogenous IL-1Ra that may contribute to prolonged inflammation and eventually neonatal diseases of the lung, heart and brain (23, 24). Moreover, in preterm fetal sheep, infusion of IL-1Ra mitigated brain inflammation and preoligodendrocyte loss that are characteristic of dWMI (22).

As anakinra, IL-1Ra has been used clinically for over 20 years, in more than 200,000 patients, including children and infants. This substantial clinical experience has demonstrated a highly favourable safety profile for anakinra (e.g (25, 26); hence, this medication, which is well established in most other age groups, represents a potential strategy for preventing or treating inflammation, including that underpinning BPD, BPD-PH and dWMI, in infants born prematurely.

Anakinra Pilot, whose full name is “A Clinical Trial to Demonstrate Safety, Feasibility and Pharmacokinetics of Interleukin 1 Receptor Antagonist in Preterm Infants”, is a Phase I/IIa trial designed to establish safety, feasibility and pharmacokinetics of early administration of anakinra to infants born <28 weeks’ gestation.

## Methods and analysis

### Primary aim

To determine safety and feasibility of administering anakinra to premature infants born between 24 weeks and 0 days (24^0^) and 27^6^ weeks of gestational age (GA) from day 1 to day 21 after birth.

### Study type

Investigator-initiated single-arm, Phase I/IIa dose-escalation safety trial.

### Site

Monash Newborn, Monash Children’s Hospital, Monash Health, Melbourne, VIC, Australia.

### Inclusion criteria

• Born between 24^0^ and 27^6^ weeks of GA
• Born at on-site at the participating hospital.

### Exclusion criteria

• Inability of the legal guardians to consent.
• Any disease or condition that the investigators judge could confound the trial results; these include, but are not limited to, genetic syndromes, severe cardiac or other abnormalities, substantial pre-/perinatal compromise (postnatal hypoxia (SaO2<80% for >3h), intrauterine stroke and others.
• Imminent death.
• Foreseeable inability to adequately excrete anakinra, i.e. severely and permanently compromised renal function.

### Secondary objectives

1. To conduct an interim pharmacokinetic analysis to compare observed concentrations with predictions from population PK simulation; and

2. To monitor changes of the plasmatic and cellular immune function conferred by treatment with anakinra.

### Sample size

We will recruit 24 infants. Given the well-established, favourable safety profile and extensive clinical experience with anakinra, including in children and infants, as well as detailed knowledge on its mechanism of action, this number of participants will suffice to demonstrate safety and feasibility. In addition, it will allow confirmation of predicted/simulated drug disposition, by comparing observed concentration with the population PK simulation, to inform dose selection for future large effectiveness trials.

### Definitions

#### Safety

For the purposes of the trial’s primary objective, safety will be defined as completion of the described treatment regimen in our target population, without any adverse events of greater frequency or severity than expected to be observed in this high-risk population. For example, at Monash Newborn the baseline rate of late-onset sepsis in this population is 27%, early-onset sepsis is 1%, death is 15%, BPD is 70% and necrotising enterocolitis is 12%.

#### Feasibility

Feasibility will be determined by successful completion of the three-week treatment course.

### Estimated duration of individual subject participation

Anakinra treatment commences at 3-24 hours of postnatal life and ceases on postnatal day 21. Treatment will continue until day 21 unless one of the following occurs: a suspected unexpected serious adverse reaction; acute kidney injury (which would increase dose-normalised exposure in the unlikely setting of immature non-renal clearance); neutropenia; or elevation of liver enzymes (details below). To monitor for these events and as part of standard care, the following blood tests will be sent to the routine pathology laboratory on days 1, 3, 7, 14, and 22 after starting IL-1Ra infusions in addition to plasmatic and cellular immunity analysis: full blood count, liver enzyme tests, urea, electrolytes and creatinine.

Treatment will cease if consent is withdrawn, in which case consent will be sought for the infant to remain enrolled and data collection to continue to enable intention-to-treat analysis. Rates of withdrawal of consent will be monitored to detect differential dropout, which can bias clinical trial results and reduce the power of the trial to detect important differences. Treatment will also cease if death is imminent and the treating team with the parents have made the decision to redirect care.

Collection of data will continue until 2 years of age, with a review at 3 months corrected age (i.e. 3 months after the infant reaches an age that equates to 40^0^ weeks GA) in the Early Neurodevelopmental Clinic (including a Prechtl’s general movements assessment), and a neurodevelopmental review at 2 years.

### Anakinra product information

Anakinra is a recombinant, non-glycosylated formulation of human IL-1Ra. It is nearly identical to the endogenous protein, the only difference being the addition of a single methionine residue at its N-terminus. The mechanism of action is through competitively inhibiting IL-1 binding to its receptor, thus preventing activation of the pro-inflammatory cascades triggered by IL-1. Anakinra has been shown to cross the blood-brain barrier in humans and preclinical models (27).

Anakinra is registered for use in various autoimmune and autoinflammatory conditions. These include rheumatoid arthritis, Still’s disease, familial Mediterranean fever, deficiency of IL-1Ra (DIRA) and cryopyrin-associated periodic syndromes (CAPS), including neonatal-onset multisystem inflammatory disease (NOMID). Typical initial dosing in adults is 100 mg daily and in children and adolescents is 1-2 mg/kg daily. Dose can be titrated to 3-4 mg/kg daily if required in 0.5-1 mg/kg increments; and in NOMID, the dose can be incremented to a maximum of 8 mg/kg daily. Population PK analysis in children and adolescents has suggested that in a child of <10 kg weight, 3 mg/kg of anakinra administered subcutaneously (SC) daily is required to achieve equivalent exposure to 1 mg/kg SC daily in an adolescent or adult (28), consistent with allometric scaling of drug clearance (29).

Anakinra undergoes extensive renal clearance, and in the setting of severe renal impairment (glomerular filtration rate, GFR <30 ml/min/1.73 m^2^), recommendation is for the same dose size to be administered alternate daily rather than daily.

Anakinra is supplied by Swedish Orphan BioVitrium and is sourced through hospital pharmacy. Anakinra is sold in ready-made syringes containing a solution (pH 6.5) comprising disodium-EDTA (0.12 mg), NaCl (5.48 mg), sodium-citrate (1.29 mg) and polysorbate 80 (0.70 mg) in water for injection. Anakinra will be provided to the ward in tuberculin syringes containing 15 mg (0.1 ml) pre-prepared by hospital pharmacy at a dilution that renders injection of the small doses required for this trial possible.

### Clinical pharmacology

Anakinra is a relatively small therapeutic protein of 153 amino acids and a molecular weight of 17.3 kDa. It is typically administered daily by SC-injection; however, it has also been administered intravenously (IV) for a range of indications. Its initial distribution volume is equivalent to plasma, with steady-state distribution volume between plasma volume and extracellular fluid volume, reflecting some extravascular movement (30).

Therapeutic proteins are eliminated almost exclusively by proteolysis, with the amino acids and peptides produced able to be reutilised in endogenous processes (31). Proteolysis of therapeutic proteins can occur via fluid-phase hydrolysis, lysosomal degradation after receptor-mediated cellular uptake or target-mediated uptake and degradation (32). In addition, therapeutic proteins with a molecular weight of less than 60 kDa, like anakinra, are freely filtered at the kidney glomerulus, with subsequent catabolism by the proximal tubular cells, either at the intraluminal brush border or after endocytosis (31).

With a molecular weight of 17.3 kDa, anakinra is efficiently filtered at the glomerulus, with renal clearance accounting for approximately 75% of total drug elimination. When dosed IV, anakinra is rapidly cleared from plasma, requiring dosing more frequently than once daily to maintain circulating anakinra concentrations throughout the dosing interval (33). However, SC-dosing of anakinra allows for less frequent dosing, due to slow absorption from the subcutaneous depot to the systemic circulation. Subcutaneous absorption is then rate-limiting to elimination, extending the duration of exposure of anakinra.

The non-renal clearance pathway for anakinra has not been described, but anakinra can be presumed to undergo endogenous proteolysis like other therapeutic proteins (32). Extra-renal clearance is lower, however, with total clearance reduction with progressive renal impairment. In a study of adults with differing levels of renal impairment, clearance of anakinra reduced with progressive renal impairment, with a reduction of 16.5%, 50.3%, 69.7% and 74.9% for GFR bands of 50-80, 30-50, <30 ml/min/1.73 m^2^ and end-stage, respectively (30). In the setting of advanced kidney disease (estimated GFR<30 ml/min/1.73 m^2^), the recommendation is to change from daily to alternate daily dosing.

### Dose selection

There are a few reports of anakinra use in early infancy (34, 35), and no reported use from birth or in extremely premature neonates. Indeed, drug development in premature neonates more broadly remains a “blind spot” in pharmacology (36). We considered dose selection a crucial element of this trial, both for safety reasons but also to ensure prospect of direct benefit to participants. Broadly accepted ethical standards for clinical research in children require potential for direct benefit if participation leads to more than minimal risk (37), and thus Phase 1 dose-finding and pharmacokinetic ‘ADME’ (absorption, distribution, metabolism and elimination) studies are not considered appropriate. Moreover, whilst in the range of auto-immune and auto-inflammatory conditions, for which anakinra is indicated, dose is titrated to clinical response, no such clinical measure for titration exists for the indication of early-life inflammation in premature infants.

Dose selection was by simulation of exposure with various dosing regimens using predicted anakinra pharmacokinetics in extremely premature neonates, in accord with standard practice in paediatric drug development. Population PK models are mathematical-statistical models providing a quantitative description of a drug’s disposition and population variability. To extrapolate to infants and neonates, PK parameters require scaling for size, and depending on the drug elimination pathways involved, a maturation function for organ immaturity. Furthermore, simulation of concentration time course and exposure with various dosing rates and schedules is performed, to determine a dose rate and schedule that matches exposure and the concentration time course that is known to be safe and effective in older children and adults. Predictions can subsequently be confirmed by comparing predictions with observed exposure, i.e. measured drug concentrations.

Data were sourced from the published literature as well as regulatory submissions, with relevant examples of observed exposure presented in **Table 1**. and pharmacokinetic parameters in **Table 2**.

**Table 1:** Example anakinra dose and exposure values from published literature.

Reference	Population	N	Dose	Route	AUC_0-24h_ (mg/lxh)	Cmax (mg/l)
Yang B et al. (30)	PD HDx Normal GFR	10 10 12	1 mg/kg	IV	63.7^a^ ( ± 9.6) 64.2 ^a^ ( ± 14.9) 9.6 ( ± 1.4)	15.6 ( ± 6.5) 14.5 ( ± 3.3) 22.4 ( ± 4.9)
Yang B 2003 (30)	GFR<30	12	100 mg	SC	39.4 ( ± 4.3)	2.2 ( ± 0.7)
Yang B et al. (38)	Adults	7	100 mg	IV	14.7 ( ± 3.4)	32.2 ( ± 6.4)
Galea et al. (27)	Adults, SAH	29	100-500 mg	IV		34-102.8 (predicted)
Urien et al. (28)	Children	87	2-10 mg/kg	SC	9.6	
Study 03-AR-0298 (39)	CAPS	21 (17 Paed)	Initial: 1 mg/kg Month 3: 1.5 mg/kg Year 3: 3 mg/kg	SC	4.4 [1.8-6.9] 7.3 40.8	

a AUC_0-48h_,Target exposure based on pharmacokinetic/pharmacodynamic analysis. Concentration data expressed as mean (± standard deviation, SD) or median [range]. CAPS, cryopyrin-associated auto-inflammatory syndromes; HDx, haemodialysis; PD, peritoneal dialysis; SAH, subarachnoid haemorrhage.

**Table 2:** Published PK and population PK parameter values for anakinra.

Reference	Population	N	Dose	Route	CL (l/h/70kg)	BSV CL (%)	V_D_ (l/70kg)
Yang B et al. (40)	Adults	341	30-150 mg	SC	6.92		9.94
Yang B et al. (30)	HD PD Normal GFR	10 10 12	1 mg/kg	IV	1.09 1.15 7.3		9.99 (initial 2.95) 9.8 (initial 3.26) 8.86 (initial 3.32)
Yang B 2003 (30)	Stratified by GFR	30	100 mg	SC	10.2 to 2.6		
Gueorguieva et al. (41)	Adult				6.96	23	5.14
Urien et al. (28)	Children	87	2-10 mg/kg	SC	6.24	54	6.52
Yang B et al. (38)	Adults Adults	32 32	100 mg 100 mg	IV SC	6.72 8.04		8.18

BSV, between-subject variability; CL, clearance; GFR, glomerular filtration rate; V_D_, volume of distribution.

There were 3 key considerations for extrapolation and simulation: size scaling of PK parameters, maturation of clearance mechanisms, and use of IV- versus SC-dosing. Modelling and simulation were performed, including between-patient variability, using the Berkeley Madonna software package (42). Graphs were rendered in R version 4.1.0 (43).

#### a) Size scaling

Size scaling of key pharmacokinetic parameters clearance (CL) and distribution volume (V_D_) was by theory-based allometry, with exponents ¾ and 1 for CL and V_D_, respectively. These are represented by equations 1 and 2:


CLi=CLSTD,70kg×(WTi70)34

VD,i=VD,STD,70kg×(WTi70)

Like many therapeutic drugs, anakinra is dosed in mg/kg in children. However, although this dosing strategy is safe and easy to implement, clearance mechanisms do not scale linearly with body weight. Provided clearance mechanisms are mature, per kilogram dosing leads to lower exposure in young (smaller) children, particularly below 2 years of age where systematic underexposure is seen (44). Two analyses of anakinra in children have confirmed lower exposure in younger children when given the same per-kilogram dose as older children and adolescents (28, 45).

Unlike per-kilogram dosing, scaling functions based on theory-based allometry, on the other hand, have both theory-based biological underpinning and extensive observational support (46). Their use is standard in drug development for small molecule drugs, for scaling across species to predict first-in-human dosing, as well as dose selection for paediatric clinical trials (47). More recently, these approaches have been shown to be predictive for therapeutic proteins as well, including down to neonatal size (32).

In a population PK analysis of anakinra in 87 children and adolescents, the authors defined an alternative scaling function for clearance (28). However, deviations from allometric scaling can relate to sampling distribution from insufficient sample size, and whilst the authors suggested that for therapeutic proteins the allometric model may not apply, subsequent literature for a range of therapeutic proteins has provided support for allometric scaling, including anakinra (32). Further, use of the scaling function in (28) would lead to substantially higher per-kilogram dosing in premature neonates (~10 mg/kg versus 3 mg/kg). We thus favoured use of the allometry, as it is both well supported and safer in our target population.

**Figure 1:**
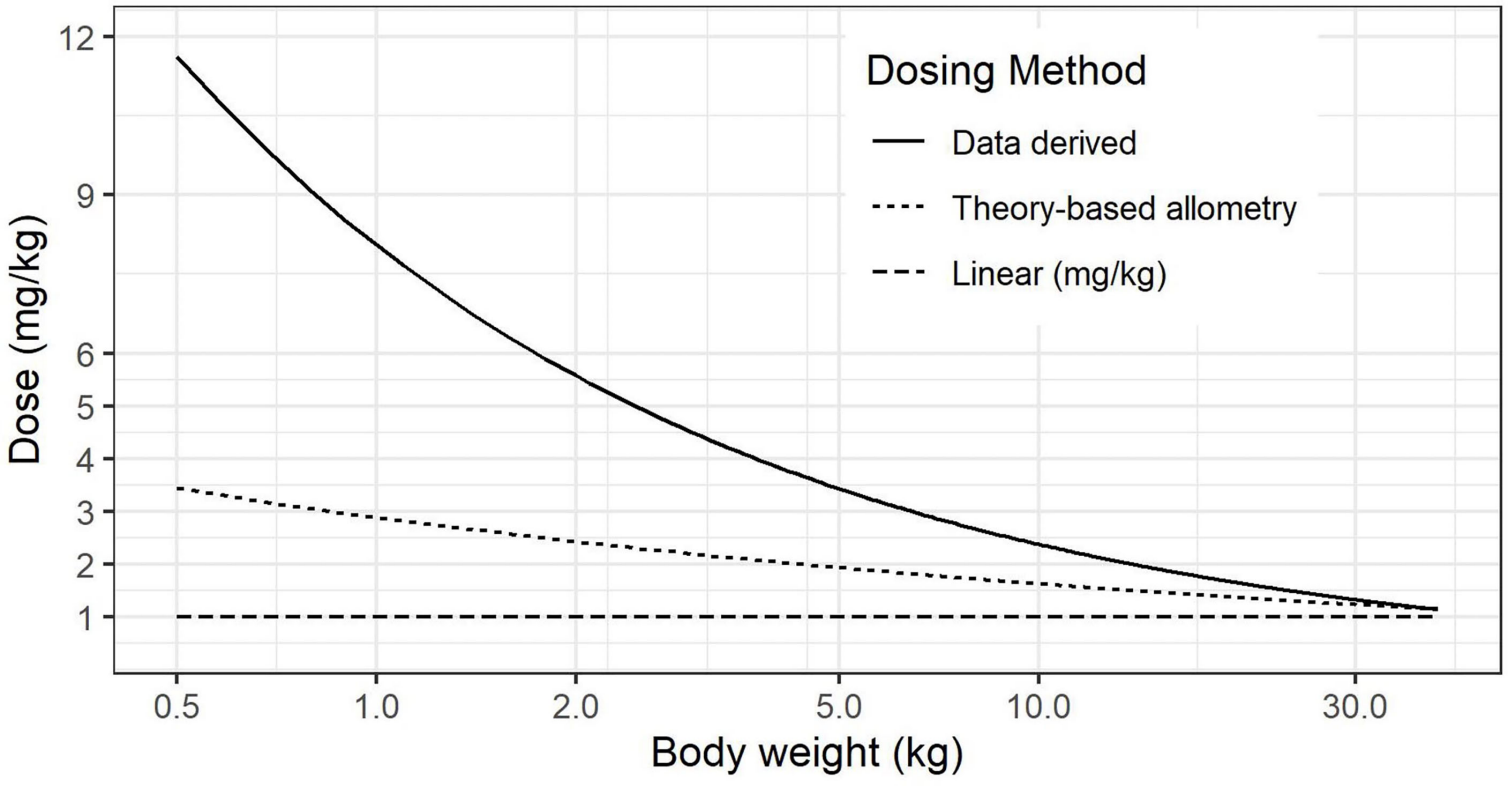
Comparison of calculated dose (mg/kg) for linear, theory-based allometry and data-driven methods (28).

The impact of size scale used on per-kilogram dosing can be seen in **Figure 1**, noting that in premature neonates in the 500 g to 2 kg weight range, there is substantial under-dosing with per-kilogram dose, along with much higher dose from (28), compared to theory-based allometry.

#### b) Maturation

After birth, renal function typically is sluggish in extremely premature neonates, with median inulin clearance expected to be <2.5 ml/min for the first 3 weeks after birth (duration of dosing) (48). Scaled for size, this is below the threshold of 30 ml/min/1.73 m^2^ (49–51), with overall clearance of anakinra thus expected to be reduced by 75% and alternate daily dosing being recommended. For prediction of renal function, a maturation model was used (52), which estimates GFR as a fraction of adult size-scaled equivalent based on post-menstrual age and size. The model was developed from a pooled population analysis of GFR (n=923) using ideal markers of GFR (polyfructose, 51Cr-EDTA, mannitol or iohexol) (52).

Non-renal clearance mechanisms of anakinra have not been described, and caution is required in new drug use in premature neonates due in part to immaturity of various hepatic drug-metabolising enzymes (53). However, no such immaturities have been described for proteolysis of therapeutic proteins (54), and indeed increased endogenous proteolysis has been described in some instances (55). Further, noting the near-identical structure to endogenous IL-1Ra, it would presumably share the same proteolytic pathways, for which there is no evidence of immaturity in the newborn period [plasma abundance of endogenous IL-1Ra in term infants was 42 ± 69 pg/ml on day 1, falling to 27 ± 77 pg/ml on day 2-3 (56)]. Nevertheless, given limited data on therapeutic proteins in extremely premature infants and thus on a precautionary basis, simulations were performed both with assumption of mature and immature non-renal clearance to better understand the impact on exposure if indeed the specific proteolytic mechanism were to be immature.

#### c) IV versus SC

SC-dosing of anakinra was not considered appropriate for the trial, given reduced subcutaneous tissue stores and skin fragility in premature neonates. Therefore, in preterm infants, IV is the preferred route of administration.

IV administration translates to substantially higher peak concentrations (38, 57). Reassuringly, there have been trials in various cohorts of very-high-dose IV anakinra therapy (e.g. 5 mg/kg and more) without any new or greater rate of toxicities seen (27, 57); it can therefore be used IV without compromising the favourable safety profile [reviewed in (58)].

A second impact of IV dosing is shorter duration of exposure, with loss of the elimination-rate limiting slow absorption from a subcutaneous depot. When administered IV to individuals with normal renal function anakinra is rapidly cleared, hence use of multiple daily dosing in trials of IV anakinra in adults (57).

**Figure 2:**
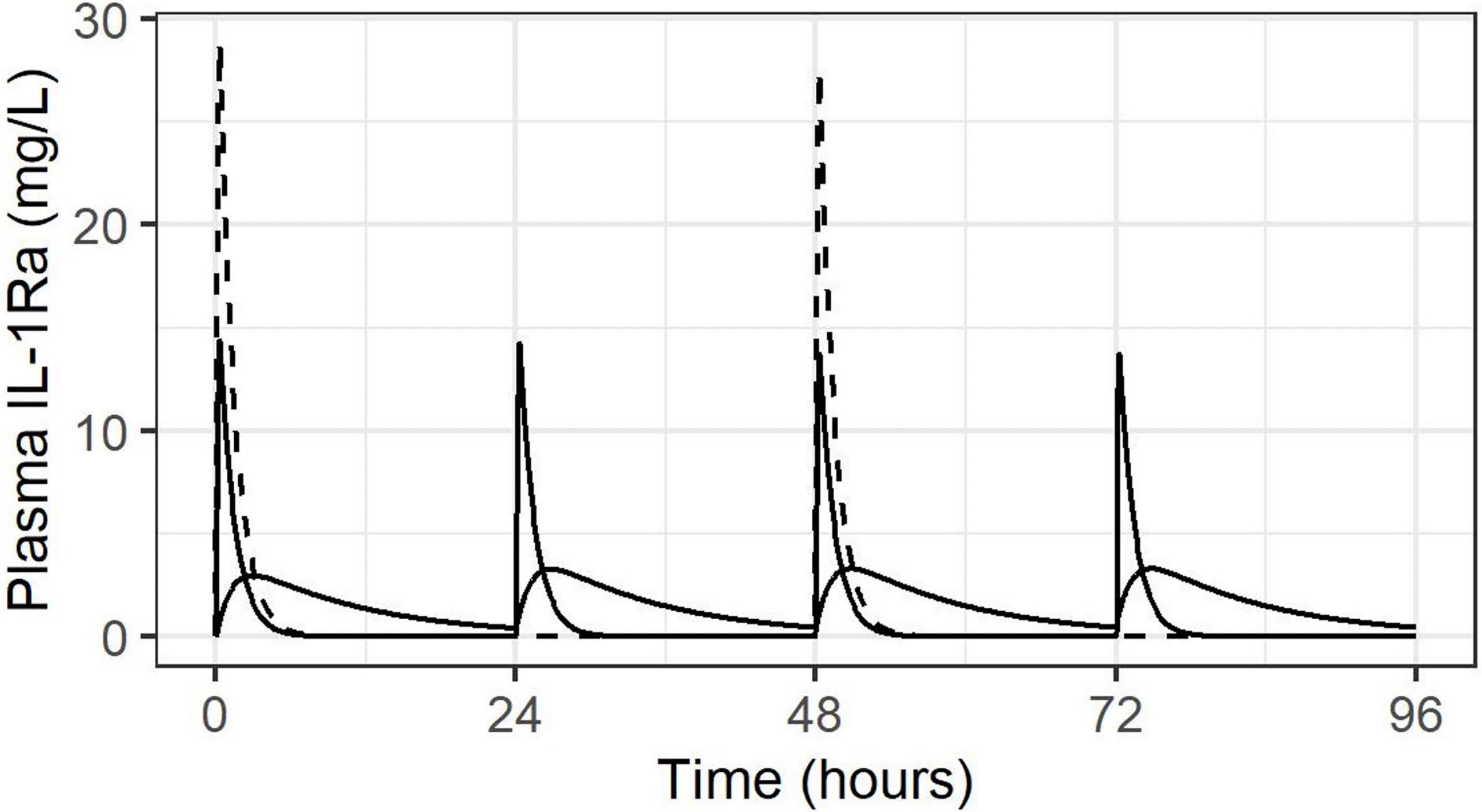
Concentration-time course following IV administration alternate daily (dashed line), or daily at 50% dose, both IV (solid line with high peaks and SC (solid line with lower, rounded peaks).

As discussed above, dosing recommendation for anakinra in the setting of severe renal impairment (as seen in our population) is for the same dose size administered alternate daily. However, to mitigate the above issues with IV administration – higher peak concentration and shorter duration of exposure – we considered administration of 50% of the dose daily. This is demonstrated schematically in **Figure 2**. With simulation of alternate daily dosing of 3 mg/kg IV, 1.5 mg/kg daily IV, and for comparison 1.5 mg/kg SC daily, in an infant born at 25 weeks GA and 850 g birth weight.

#### d) Simulations

Initial dose recommendation based on product label is for 1 mg/kg daily, though in the setting of GFR<30 ml/min/1.73 m^2^, the same dose would be administered alternate daily. At the same time, allometric scaling would suggest a per-kilogram dose of 3 mg/kg daily in a neonate weighing 500g to 1 kg to give equivalent exposure to 1 mg/kg in a 70 kg adolescent. In the setting of GFR<30 ml/min/1.73 m^2^, this would equate to 3 mg/kg alternate daily or, based on above considerations for IV dosing,1.5 mg/kg daily.

Various dosing regimens were simulated, using population PK parameters of anakinra, population parameter variability, and scaling for size and maturation of renal function. In addition, simulations were performed with 50% and 75% reduction in non-renal clearance, to assess impact on exposure. For each dosing regimen and population, 1000 simulations were performed and compared to literature values. Based on simulations and literature comparisons, notably including a threshold of 9.6 mg/l x h identified as the exposure above which sufficient suppression in auto-immune disease is achieved (28), a dose of 1.5 mg/kg daily was deemed the optimal dosing regimen.

**Figure 3:**
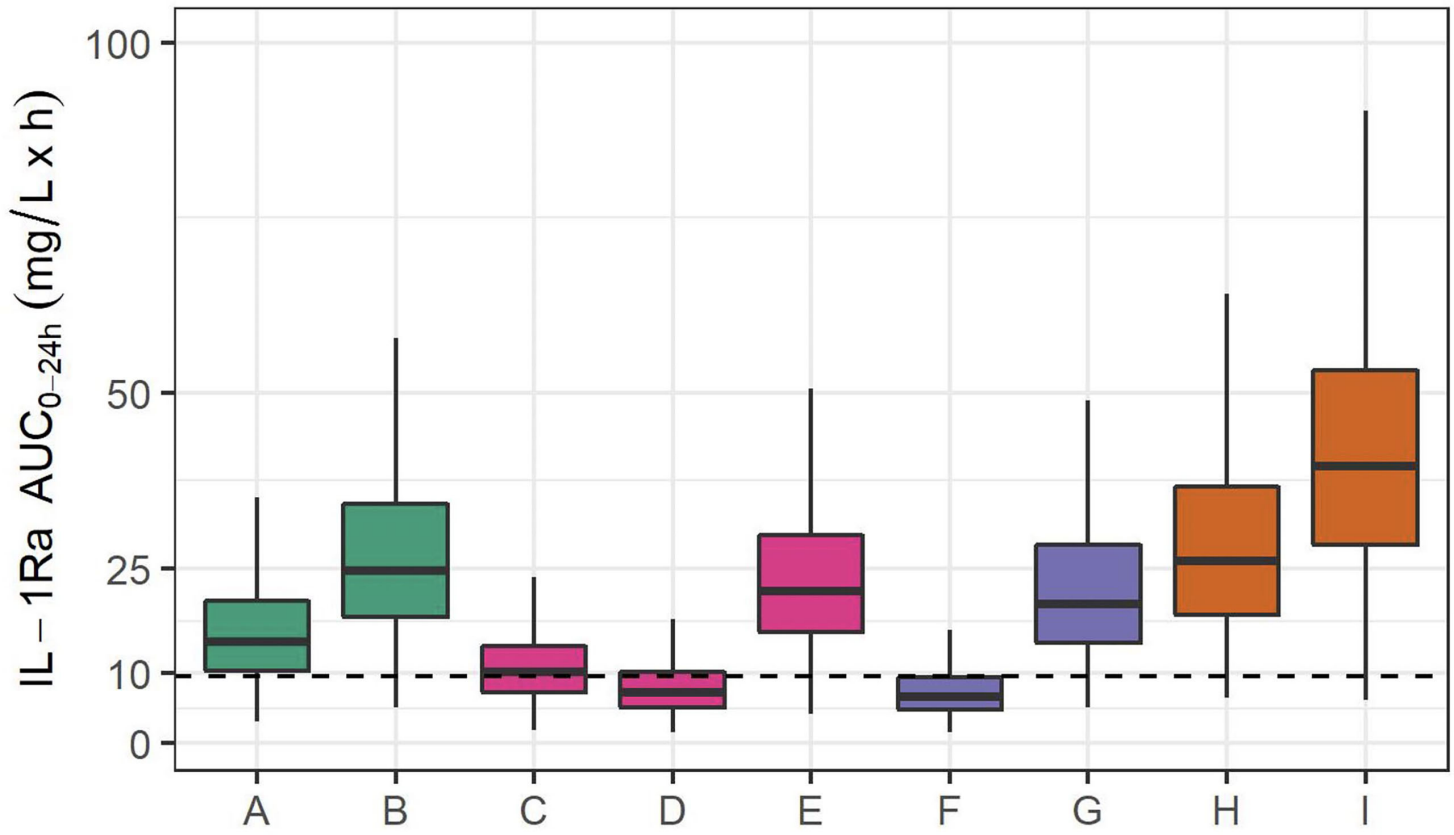
IL-1Ra plasma exposure (AUC_0-24_) from 1000 simulations, in studied populations and extrapolated to extremely premature neonates. Simulated exposure for **(A)** a 70 kg adult receiving 100 mg daily, **(B)** a 70 kg adult with severe renal impairment receiving 100 mg daily, **(C)** a 70 kg adolescent receiving 1 mg/kg daily, **(D)** a 2-year-old child weighing 12 kg and receiving 1 mg/kg daily, **(E)** a 12 kg 2-year-old child receiving 3 mg/kg daily. Simulations in extrapolated PK: **(F)** an extremely premature neonate (25-weeks GA, 850 g) with mature non-renal clearance receiving 1 mg/kg alternate daily, **(G)** an extremely premature neonate receiving 1.5 mg/kg daily, **(H)** an extremely premature neonate with 25% function of non-renal clearance receiving 1 mg/kg alternate daily, and **(I)** an extremely premature neonate with 25% function of non-renal clearance receiving 1.5 mg/kg daily. The dashed line indicates the threshold of 9.6 mg/l x h identified in (28) as the exposure above which sufficient suppression in auto-immune disease is achieved.

However, if this predicted optimal dosing rate of 1.5 mg/kg daily were used from the start and non-renal clearance were immature, this dosing rate would lead to excessive exposure, beyond that seen in older children receiving 3 mg/kg daily of anakinra. This eventuality is avoided with the planned staged approach, in which the first 6 participants receive 1 mg/kg alternate daily, followed by interim PK analysis. The rationale for this decision is as follows. Keeping the threshold of 9.6 mg/l x h in mind beyond which sufficient anti-inflammatory activity can be expected (28), **Figure 3** depicts simulations in studied populations (A-E) and extrapolated populations (F-I), with F and G depicting the predicted exposure if non-renal clearance is fully mature, H and I if non-renal clearance is only 25% mature. Assuming mature non-renal clearance, the stage 1 dosing regimen of 1 mg/kg alternate daily predicts exposure equivalent to 1 mg/kg in a child weighing 12 kg, whilst Stage 2 (optimal) dosing of 1.5 mg/kg daily predicts exposure equivalent to 3 mg/kg in a child of 12 kg. Interim analysis after the first six participants will allow confirmation of non-renal clearance being mature, and enable safe progression to the 1.5 mg/kg daily dosing schedule. On the other hand, in the unlikely case that non-renal clearance is immature (for example at 25% maturity), the initial dosing regimen of 1 mg/kg alternate daily will lead to exposure close to that seen with dosing rate of 3 mg/kg daily in an older child. If non-renal clearance were found to be immature at interim analysis, dosing in Stage 2 would be adjusted accordingly.

**Figure 4:**
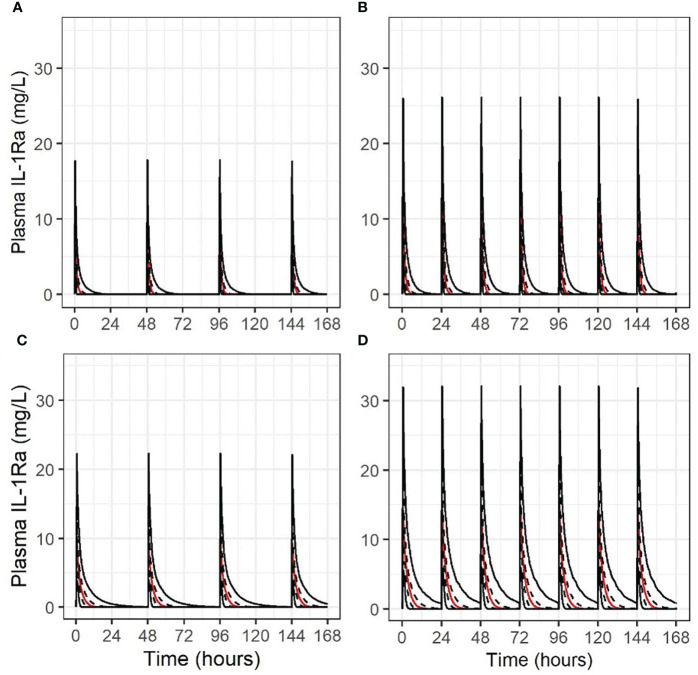
Concentration-time course for 1000 simulations (2.5^th^/25^th^/50^th^/75^th^/97.5^th^ centiles, indicated by solid, dashed, red, dashed and solid lines in this order, respectively), for different scenarios of anakinra IV dosing in an extremely premature neonate. **(A)** 1 mg/kg alternate daily, mature non-renal clearance; **(B)** 1.5 mg/kg daily, mature non-renal clearance; **(C)** 1 mg/kg alternate daily, non-renal clearance at 25% maturity; **(D)** 1.5 mg/kg daily, non-renal clearance at 25% maturity.

The concentration-times for the 4 scenarios are shown in **Figure 4**. i.e. Stage 1 and 2 dosing with mature (**Figures 4A, B**), then immature non-renal clearance (**Figures 4C, D**). Maximum concentrations are consistent with published experience, and accumulation only occurs in a limited fashion in the Stage 2 regimen with immature non-renal clearance, not in any other scenario.

### Summary dosing strategy

For Stage 1, six infants will receive 1.0 mg/kg IV given alternate daily from day 1 to day 21 after birth. In the event of acute kidney injury (AKI), detection during blood monitoring within the 3 weeks of treatment, anakinra treatment will be halted until renal function has recovered as defined below.

After completion of Stage 1, the Investigators and the data safety monitoring board (DSMB) will conduct an interim analysis, including assessment of mortality and rate of complications of prematurity, and comparison of observed exposure with the predicted exposure estimated from the population PK simulation. If appropriate, with confirmation of mature non-renal clearance within the first cohort of 6 infants, Stage 2 will administer 1.5 mg/kg per day for the remaining 18 infants. Furthermore, dosing will not need to be withheld in the setting of acute kidney injury (AKI) if non-renal clearance is confirmed to be mature from birth.

### Resulting overall protocol algorithm

**Figure 5:**
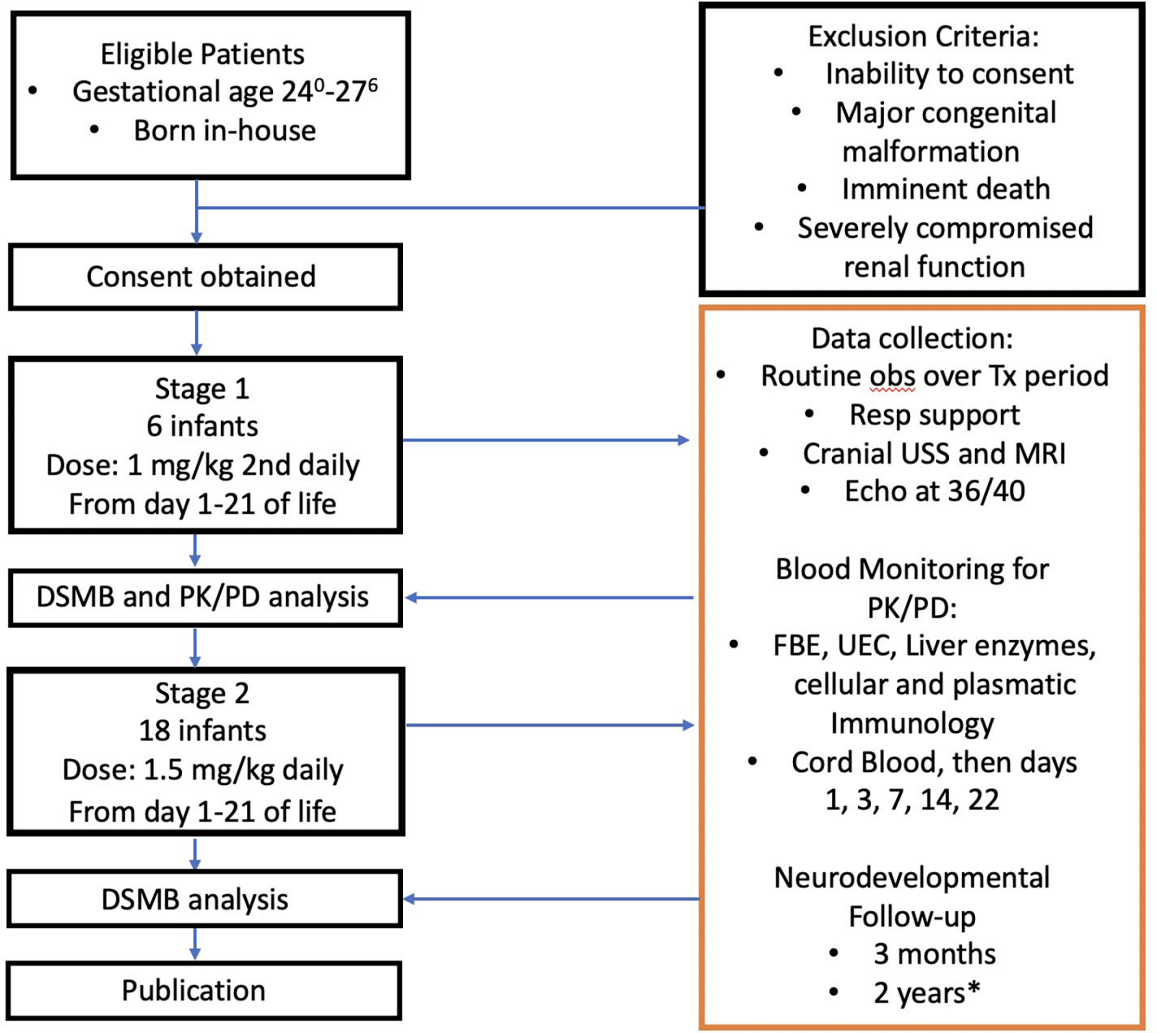
Anakinra Pilot flow chart. DSMB, Data Safety Monitoring Board; USS, ultrasound study; MRI, magnetic resonance imaging; PK/PD, pharmacokinetic/pharmacodynamic analysis; FBE, full blood examination; UEC, urea, electrolytes, creatinine. *, Publication will not await these result, 2 year follow-up results will be published separately.

A flow chart illustrating the protocol resulting from the considerations discussed above is shown in **Figure 5**.

### Indications for interruptions of treatment

Treatment will continue until day 21 unless one of the following occurs: a suspected unexpected serious adverse reaction (SUSAR); acute kidney injury, which will increase dose-normalised exposure if immature nonrenal clearance is demonstrated in Stage 1; neutropenia; or elevation of liver enzymes. To monitor for these events, the following blood tests will be sent to hospital pathology on days 3, 7 and 14 after birth: FBC, ALP, ALT, UEC.

#### SUSAR

Treatment may re-commence upon conclusion of the deliberations on causality of the SUSAR.

#### Acute kidney injury (AKI)

AKI will be defined as a serum creatinine > 120 µmol/l (59) or anuria (<0.1 ml/kg/h) for more than 24 h (60). Such AKI will necessitate temporary stopping of anakinra administration. Administration can be recommenced once renal function improves (serum creatinine < 120 µmol/l and urine output >0.5 ml/kg/h for at least 24 h). The need to withhold anakinra following AKI will be reassessed after analysis of Stage 1 (n=6 infants), including anakinra plasma concentration data, noting that the recommended dose adjustment in children and adults is alternative daily, in all degrees of renal impairment less than 30 ml/min/1.73 m^2^.

#### Drug-induced liver injury

Drug-induced liver injury will be monitored for as described above and responded to as follows:

1. An increase in ALT to greater than 3x upper limit of normal (ULN) requires repeat within 48 h, including INR.
2. Anakinra should be held if ALT >5x ULN.
3. Anakinra will be permanently ceased if:
ALT >8x ULN
ALT >5x ULN for 2 weeks
ALT >3x ULN and INR>1.8
ALT >3x ULN with eosinophilia (>5%) (61)

Anakinra can be restarted when ALT fall <3x ULN.

#### Serious infection

Anakinra at higher doses (>100 mg SC daily in adults) has been associated with an increased risk of development of infection (25). However, whether or not it is sensible to withhold anakinra in an individual who develops an infective episode is unclear. Anakinra has been used in large randomised controlled trials of sepsis (>1000 patients) as well as syndromes associated with cytokine storm, showing some evidence of benefit and no signal of increased morbidity/mortality (62). In the sepsis trials, anakinra was given over the initial 72 h (57), thus safety in the setting of infection beyond 72 h cannot be assumed. In the event of a positive blood culture and clinical signs of sepsis, anakinra will be withheld if the neutrophil count is <0.5 x 10^9^/l and/or circulatory support with infusion of inotropic medications is required and cannot be weaned 48 h after commencement. Anakinra can be recommenced with recovery of the neutrophil count to >0.75 x 10^9^/l or cessation of inotropic treatment.

### Safety monitoring

All participating infants will be monitored closely following the infusion of anakinra as is standard clinical practice at Monash Children’s Hospital’s Neonatal Intensive Care Unit (NICU) and most other NICUs, with particular attention to vital signs in the 10 min immediately after the administration of anakinra. Adverse event (AE) reporting will be commensurate with the clinical course expected for the study population of extremely preterm infants.

Adverse Events are defined as:

• Any untoward medical occurrence in a patient enrolled into the study
• Sustained unexpected change of 30% or more from baseline vital signs (heart rate, blood pressure, respiratory rate, oxygen saturation, body temperature) within 10 min of anakinra administration
• Unexpected need to escalate respiratory support within 12 h of anakinra administration
• Unexpected need to initiate or escalate inotropic support within 12 h of anakinra administration
• Any event requiring cardiopulmonary resuscitation that occurs within 12 h of anakinra administration will be investigated as a potential serious AE.

To assess safety, the following monitoring will be conducted:

1. Early respiratory outcomes will comprise mode of ventilation, lowest pH, highest pCO_2_, highest FiO_2_ and highest mean airway pressure or continuous positive airway pressure (CPAP) level. The respiratory index will be calculated from the last 2 items. Each of these parameters will be recorded daily for the entire treatment duration, and on postnatal day 42. We will also record:

a) each intubation event up to postnatal day 42, including the reason for intubation (e.g. FiO_2_ requirement, respiratory acidosis, apnoea, other);
b) surfactant administration (number of doses);
c) administration of inhaled nitric oxide (yes/no);
d) maximum maintenance caffeine dose used (mg/kg/day).

For analysis purposes, we will further record:

e) Maternal clinical data from the ante- and peri-natal period, including duration of rupture of membranes, maternal fever, chorioamnionitis, antibiotic therapy, maternal medication (including antenatal steroids and magnesium sulfate), hypertension, gestational diabetes, abnormalities of antenatal ultrasounds, and other conditions relevant to the wellbeing of the infant; and
f) mode of delivery, Apgar score and resuscitation.

2. Later cardiopulmonary outcomes will comprise duration/last day receiving of:

a) mechanical ventilation;
b) respiratory support including CPAP and high flow;
c) supplementary oxygen.
d) Moreover, we will record total days of mechanical ventilation, high frequency oscillation and postnatal steroids; BPD [yes/no, Jensen grade (63)]; pulmonary hypertension (yes/no by echocardiography at ~36 weeks corrected GA); and discharge to other hospital or home on respiratory support or oxygen.

3. From blood tests obtained for clinical reasons, in addition to the parameters already mentioned above, the following will also be recorded: Full blood counts, urea, electrolytes, creatinine, liver enzyme studies, blood cultures and viral swabs. Monitoring of cellular and plasmatic immunology will be conducted on blood samples and of the microbiome on stool and respiratory samples.

4. From the clinical charts, we will monitor the occurrence of sepsis, necrotising enterocolitis, intracranial haemorrhage and medications.

5. Infants will receive cranial ultrasounds according to standard monitoring protocol on days 3, 8, 14 and 42 after birth. They will also have a magnetic resonance scan (MRI) of the brain at term-equivalent age, i.e. at 38-42 weeks GA, in which brain structure, and presence or absence of dWMI will be assessed in detail. For this purpose, a scoring system will be used wherein white matter will be graded on a scale of 0-4 for the following six variables: 1) cystic degeneration, 2) focal signal abnormalities, 3) delayed myelination, 4) thinning of the corpus callosum, 5) dilated lateral ventricles, and 6) reduction of WM volume as per (64).

6. Although not part of the trial’s primary safety outcomes, early neurodevelopmental assessments will be undertaken in the neonatal period [General Movement Assessment (GMA-writhing period (65)], Hammersmith neonatal neurological examination (HNNE) (66), orthopaedic assessment (plagiocephaly, hip dysplasia) and screening for early diagnosis of cerebral palsy and significant motor delay at 3-4 months corrected age (GMA-fidgety period, Hammersmith infant neurological examination (HINE) (67), orthopaedic and infant feeding screening assessments) in the Monash Newborn Early Neurodevelopment Clinic.

### Blood sampling procedure

Central or peripheral catheters will be used whenever possible. If no catheter is available, blood will be obtained by heelprick or venipuncture performed for routine diagnostic blood sampling whenever possible. Thus, exposure of the patients to painful procedures outside routine clinical care is minimised. We obtain 0.5-1 ml of blood (0.5 ml if patient weight is currently <1 kg, 0.8 ml if 1-2 kg, 1 ml if >2 kg) for each sampling timepoint to perform PK and cellular and plasmatic immune analysis on each sample.

PK blood samples will be taken:

1. From cord blood (baseline)
2. On days 1, 3, 7 and 14 of postnatal life. These samples will be collected within 21-23 h post anakinra administration
3. 21-23 h after the last dose of anakinra, i.e. on day 22.

### Statistics - clinical

Continuously collected physiological data (e.g. heart rate, SpO_2_, respiratory rate) will be summarised as area under the curve (AUC) in 24 h epochs. A cumulative AUC for the first 21 days will be calculated for each participant, and the mean ((± standard deviation, SD) of this variable compared between the two dosages (1.0 mg/kg 48-hourly in Stage 1 and up to 1.5 mg/kg 24-hourly in Stage 2).

### Duration of trial

Assuming rates of admission do not change dramatically, and a recruitment rate of 20-25% of eligible babies, we will be able to enrol 8-15 patients per year; hence, we expect completion of enrolment in 24 months.

## Ethics and dissemination

### Data Safety Monitoring Board

An independent DSMB has been formed, comprising three independent neonatologist researchers, to review adverse incidents and the PK analysis. A review will be undertaken after the first 3 and 6 infants have completed treatment. After the first 6 infants, the Investigators and the DSMB will agree on Stage 2 dosing.

### Reporting to the Human Research Ethics Committee

The following will be reported to the HREC:

• Any severe adverse event thought to be related in any way to the study
• Annual research progress report
• Any updates to the protocol or Participant Information and Consent From (PICF)

### Ethics

This study is being conducted in compliance with the approved protocol/amendments, conditions of Monash Health HREC approval and the NHMRC National Statement on Ethical Conduct in Human Research. Monash Health HREC granted ethics approval on 10^th^ January 2022, RES-21-0000-681A. The steering committee and hospital ethics committee deliberated on inclusion criteria and elected to define the lower GA inclusion limit of 24 weeks due to the high rates of poor outcomes in infants born<24 weeks GA, and the already high emotional burden on these families.

### Consent

Fully informed and written consent will be obtained antenatally whenever possible, but may be obtained up to 23 h after birth, after a detailed discussion of the risks and benefits of this study. In all cases, written consent will be obtained using a specifically designed PICF. Participation will be voluntary and withdrawal possible at any stage.

### Dissemination

Results of this trial will be submitted for publication in a peer-reviewed journal, and dissemination in lay media to inform consumers and the general public will be sought.

## Data availability statement

The original contributions presented in the study are included in the article/supplementary materials. Further inquiries can be directed to the corresponding authors.

## Ethics statement

The studies involving human participants were reviewed and approved by Human Research and Ethics Committee Monash Health. Written informed consent to participate in this study was provided by the participants’ legal guardian/next of kin.

## Author contributions

Study foundations and concept: PB, CN-P, MN. Detailed study design: all authors. PK/PD modelling and study design: DM, CK. Obtained funding: RG, RH, CN-P, MN. Original draft of AP’s investigator brochure: PB, CN-P, MN. Contributed to current version of AP’s investigator brochure: all authors. Wrote this manuscript: all authors All authors contributed to the article and approved the submitted version.

## Funding

Investigators were supported by an NHMRC Investigator Grant 1173584 to CN-P, the Fielding Foundation Fellowship 2017 (to MN), the Cerebral Palsy Alliance PhD Scholarship 2022 to EG, the CJ Martin Postdoctoral Fellowship and grants from the National Health and Medical Research Council of Australia (1090890 and 1164954), the Cerebral Palsy Alliance to RG, Harold and Cora Brennen Benevolent Trust, Health Research Council of New Zealand (17/601) to AG, and by the Victorian Government’s Operational Infrastructure Support Program.

## Acknowledgments

The authors acknowledge the contributions of Anakinra Pilot’s associate investigators, including in alphabetical order Dr. Steven Cho, Professor Michael Ditchfield, Associate Professor Atul Malhotra Dr. Pramod Pharande, Professor Graeme Polglase, Dr. Calum Roberts, Dr. Ina Rudloff, and Associate Professor Kenneth Tan. We are also extremely grateful for the support of Anakinra Pilot by the infant participants and their families, and by the medical, nursing and allied health teams at Monash Children’s Hospital and Monash Newborn.

## Conflict of interest

The authors declare that the research was conducted in the absence of any commercial or financial relationships that could be construed as a potential conflict of interest.

The handling editor GT declared a shared parent affiliation with the author AG at the time of review.

## Publisher’s note

All claims expressed in this article are solely those of the authors and do not necessarily represent those of their affiliated organizations, or those of the publisher, the editors and the reviewers. Any product that may be evaluated in this article, or claim that may be made by its manufacturer, is not guaranteed or endorsed by the publisher.
